# Resistance status of lepidopteran soybean pests following large-scale use of MON 87701 × MON 89788 soybean in Brazil

**DOI:** 10.1038/s41598-021-00770-0

**Published:** 2021-10-29

**Authors:** Renato J. Horikoshi, Oderlei Bernardi, Daniela N. Godoy, Altair A. Semeão, Alan Willse, Gustavo O. Corazza, Elderson Ruthes, Davi de S. Fernandes, Daniel R. Sosa-Gómez, Adeney de F. Bueno, Celso Omoto, Geraldo U. Berger, Alberto S. Corrêa, Samuel Martinelli, Patrick M. Dourado, Graham Head

**Affiliations:** 1 Bayer Crop Science, Santa Cruz das Palmeiras, SP Brazil; 2 Departamento de Defesa Fitossanitária, Universidade Federal de Santa Maria, Santa Maria, Rio Grande Do Sul, RS Brazil; 3 Regulatory Science, Bayer Crop Science US, Chesterfield, MO USA; 4 Fundação ABC, Castro, PR Brazil; 5 Departamento de Entomologia e Acarologia, Escola Superior de Agricultura “Luiz de Queiroz”, Universidade de São Paulo, Piracicaba, SP Brazil; 6 Empresa Brasileira de Pesquisa Agropecuária - Embrapa Soja, Londrina, Paraná, PR Brazil; 7 Bayer Crop Science, São Paulo, SP Brazil

**Keywords:** Entomology, Environmental economics

## Abstract

Widespread adoption of MON 87701 × MON 89788 soybean, expressing Cry1Ac *Bt* protein and glyphosate tolerance, has been observed in Brazil. A proactive program was implemented to phenotypically and genotypically monitor Cry1Ac resistance in *Chrysodeixis includens* (Walker). Recent cases of unexpected injury in MON 87701 × MON 89788 soybean were investigated and a large-scale sampling of larvae on commercial soybean fields was performed to assess the efficacy of this technology and the distribution of lepidopteran pests in Brazil. No significant shift in *C. includens* susceptibility to Cry1Ac was observed eight years after commercial introduction of this technology in Brazil. F_2_ screen results confirmed that the frequency of Cry1Ac resistance alleles remains low and stable in *C. includens*. Unexpected injury caused by *Rachiplusia nu* (Guenée) and *Crocidosema aporema* (Walsingham) in MON 87701 × MON 89788 soybean was detected during the 2020/21 season, and studies confirmed a genetically based alteration in their susceptibility to Cry1Ac. MON 87701 × MON 89788 soybean remains effective against *Anticarsia gemmatalis* (Hübner), *C. includens*, *Chloridea virescents* (Fabricius) and *Helicoverpa armigera* (Hübner) in Brazil. However, there is evidence of field-evolved resistance to MON 87701 × MON 89788 soybean by the secondary soybean pests *R. nu* and *C. aporema*.

## Introduction

Pest management in commercial fields of soybean [*Glycine max* L. (Merr.)] in South America has been transformed by the introduction of MON 87701 × MON 89788 soybean biotechnology (commercial name Intacta RR2 PRO). MON 87701 × MON 89788 expresses the Cry1Ac (event MON 87701) protein from *Bacillus thuringiensis* (*Bt*) var. *kurstaki* at high levels all season long^1,2^, and tolerance to glyphosate is conferred by the event MON 89788, which expresses CP4 EPSPS. Brazil was the first country to adopt MON 87701 × MON 89788 soybean in 2013/14. Eight years after its first commercial cultivation, more than 30 million hectares have been cultivated with this technology in Brazil (80% of the country’s soybean acreage) in the 2020/21 crop season^3^.

The high adoption rate of MON 87701 × MON 89788 soybean has been driven by the significant yield advantage of varieties containing this technology and the high levels of protection against the primary soy lepidopteran pests in Brazil. MON 87701 × MON 89788 soybean provides effective protection against larval feeding by *Anticarsia gemmatalis* (Hübner, 1818) (Lepidoptera: Erebidae), *Chrysodeixis includens* (Walker, [1858]), *Chloridea virescens* (Fabricius, 1781) and *Helicoverpa armigera* (Hübner, 1808) (Lepidoptera: Noctuidae)^4–7^.

The development of resistance in populations of the target pests to transgenic plants expressing *Bt* insecticidal proteins has been the main threat to the sustainability of these technologies^8,9^. The refuge strategy has been recommended as the major insect resistance management (IRM) strategy for delaying resistance in target pest populations of MON 87701 × MON 89788 soybean^1,4,6,10^. The assumption is that a *Bt* plant expressing a high dose of the *Bt* protein would control most or all of the individuals heterozygous for resistance alleles in natural populations, making resistance functionally recessive. When a sufficient refuge of non-*Bt* plants is available near a *Bt* field, the rare homozygous resistant insects that survive on *Bt* plants mate with the relatively abundant homozygous susceptible insects in the refuge. As a result, the heterozygous offspring produced would not survive on the high-dose *Bt* crop, substantially delaying the evolution of resistance^11^. The technical refuge recommendation for MON 87701 × MON 89788 soybean in Brazil is planting at least 20% of the soybean acreage as a structured refuge formed by non-*Bt* soybean and located within 800 m of the *Bt* crop area^12^. However, low compliance with structured refuge recommendations seems to be a common theme across most of the cases of insect resistance to *Bt* crops documented globally^13–21^. This highlights the importance of programs to monitor the development of insect resistance to MON 87701 × MON 89788 soybean and assess the adoption of refuge planting according to the technical recommendations for this technology in Brazil.

In addition to protecting against the insect species already mentioned, MON 87701 × MON 89788 soybean also offers protection against larval injury caused by *Rachiplusia nu* (Guenée, 1852) (Lepidoptera: Noctuidae) and *Crocidosema aporema* (Walsingham, 1914) (Lepidoptera: Tortricidae)^1^. *Rachiplusia nu* and *C. aporema* are historically considered key soy lepidopteran pests in Argentina^22–24^; in Brazil, however, the occurrence of these species in soybean fields has historically been restricted to reasonably low levels in the mid-south region of the country^25–27^. Cases of unexpected injury to the MON 87701 × MON 89788 soybean caused by “loopers” were identified during the 2020/21 crop season. We investigated these cases and here we report that the insects causing unexpected injury were *R. nu* and *C. aporema*.

The goals of this paper are to (a) report data from the proactive resistance monitoring of *C. includens* to MON 87701 × MON 89788 soybean in Brazil; (b) characterize the level of Cry1Ac resistance in *R. nu* and *C. aporema* samples collected during the 2020/21 season; and (c) assess the efficacy of the MON 87701 × MON 89788 technology and abundance of key lepidopteran pests in the 2020/21 season.

## Methods

### Proactive MON 87701 × MON 89788 soybean resistance monitoring of *C. includens*

#### Insect samples and permits

To proactively monitor resistance to Cry1Ac protein expressed in *Bt* soybean, *C. includens* larvae were sampled from commercial plantings of non-*Bt* soybeans in distinct geographic regions of Brazil and sent to a laboratory in our monitoring network, where they were reared on artificial diet^28^ to obtain F_1_ and F_2_ generation larvae for use in bioassays. All *C. includens* field populations sampled in Brazil are listed in Tables 1, 2, 3 of the Supporting Information (SI). All insect collections in Brazil were done in accordance with the approval granted by the System of Authorization and Information on Biodiversity (SISBIO) of the Ministry of Environment to a contracted company responsible for the field sampling (PROMIP, Permit for scientific purpose activity: 61826 and 61824).

**Table 1:** Frequency of resistance alleles conferring resistance of *C. includens* to MON 87701 × MON 89788 soybean in Brazil from 2016/17 to 2020/21.

Location	Number of tested		Number of survivors		F_2_ lines surviving at 4 days	F_2_ lines surviving at pupa stage	Resistance allele frequency	95% CI
	F_2_ lines	Larvae	Larvae 4d	Pupa				
**2016/17 soybean season**								
Sapezal, MT	50	3,408	0	0	0	0	0.0048	0.0001–0.0177
Uberlândia, MG	52	5,920	2	1	2	1	0.0139	0.0029–0.0332
Casa Branca, SP	44	6,048	2	1	1	1	0.0109	0.0013–0.0301
Campo Verde, MT	37	5,577	1	0	1	0	0.0128	0.0076–0.0162
Luís Eduardo Magalhães, BA	95	12,066	15	11	6	6	0.0180	0.0073–0.0335
Correntina, BA	111	15,104	18	13	11	9	0.0265	0.0138–0.0432
Campo Mourão, PR	89	9,840	0	0	0	0	0.0027	0.0001–0.0101
Campo Grande, MS	13	1,984	5	2	2	2	0.0501	0.0107–0.1173
Não-Me-Toque, RS	57	7,360	0	0	0	0	0.0043	0.0001–0.0156
Rolândia, PR	36	4,224	3	1	3	1	0.0264	0.0073–0.0570
*Brazil (2016/17)*	*584*	*71,531*	*46*	*29*	*26*	*20*	*0.0115*	*0.0076–0.0162*
**2017/18 soybean season**								
Campo Grande, MS	50	8,480	0	0	0	0	0.0048	0.0001–0.0178
Campo Verde, MT	50	11,232	0	0	0	0	0.0048	0.0001–0.0178
Londrina, PR	62	13,664	0	0	0	0	0.0039	0.0001–0.0144
Luís Eduardo Magalhães, BA	81	13,408	0	0	0	0	0.0030	0.0001–0.0111
Rio Verde, GO	58	6,944	0	0	0	0	0.0042	0.0001–0.0154
Campo Mourão, PR	18	2,560	5	4	1	1	0.0251	0.0031–0.0687
Cristalina, GO	27	4,152	0	0	0	0	0.0084	0.0002–0.0307
Dourados, MS	28	4,832	0	0	0	0	0.0084	0.0002–0.0307
Ponta Grossa, PR	48	7,578	14	0	4	0	0.0250	0.0082–0.0507
Casa Branca, SP	33	4,128	0	0	0	0	0.0072	0.0002–0.0263
Uberlândia, MG	37	4,128	1	0	1	0	0.0129	0.0016–0.0355
Não-Me-Toque, RS	20	2,976	0	0	0	0	0.0115	0.0003–0.0417
Bagé, RS	10	1,088	0	0	0	0	0.0210	0.0005–0.0759
*Brazil (2017/18)*	*522*	*85,170*	*20*	*4*	*6*	*1*	*0.0033*	*0.0013*–*0.0062*
**2018/19 soybean season**								
Correntina, BA	169	36,160	12	7	3	1	0.0058	0.0016–0.0128
Luís Eduardo Magalhães, BA	78	12,768	2	0	2	0	0.0093	0.0094–0.0225
Roda Velha, BA	22	4,480	0	0	0	0	0.0104	0.0003–0.0383
Cristalina, GO	62	12,112	3	3	2	2	0.0117	0.0024–0.0281
Rio Verde, GO	58	13,120	0	0	0	0	0.0042	0.0001–0.0154
Tasso Fragoso, MA	92	21,280	5	2	2	2	0.0027	0.0001–0.0098
Campo Grande, MS	117	44,582	21	15	6	6	0.0126	0.0046–0.0244
Maracaju, MS	76	16,448	0	0	0	0	0.0032	0.0001–0.0118
Campo Verde, MT	30	4,292	0	0	0	0	0.0078	0.0002–0.0287
Campo Mourão, PR	108	24,224	6	1	4	0	0.0114	0.0037–0.0231
Londrina, PR	127	29,312	1	0	1	0	0.0039	0.0005–0.0108
Passo Fundo, RS	17	3,028	1	1	1	1	0.0264	0.0033–0.0723
Casa Branca, SP	90	19,056	3	3	2	2	0.0082	0.0017–0.0196
Chapadão do Sul, MS	36	4,384	0	0	0	0	0.0066	0.0002–0.0243
Lucas do Rio Verde, MT	44	6,800	0	0	0	0	0.0055	0.0001–0.0201
Rondonópolis, MT	49	8,912	0	0	0	0	0.0049	0.0001–0.0181
Luís Eduardo Magalhães, BA	55	10,464	10	0	6	0	0.0044	0.0001–0.0162
*Brazil (2018/19)*	*1230*	*271,422*	*64*	*29*	*29*	*12*	*0.0061*	*0.0041*–*0.0084*
**2019/20 soybean season**								
Campo Verde, MT	86	20,128	0	0	0	0	0.0028	0.0001–0.0105
Luís Eduardo Magalhães, BA	144	30,256	14	2	3	1	0.0068	0.0018–0.0149
Rio Verde, GO	99	21,696	0	0	0	0	0.0025	0.0001–0.0092
Campo Mourão, PR	113	16,363	9	0	3	0	0.0087	0.0024–0.0190
Correntina, BA	129	20,572	19	12	1	1	0.0038	0.0005–0.0106
Maracaju, MS	112	18,226	5	4	2	2	0.0066	0.0014–0.0158
Campo Grande, MS	67	17,912	0	0	0	0	0.0036	0.0001–0.0134
Cascavel, PR	63	15,328	1	0	1	0	0.0077	0.0009–0.0214
Chapadão do Sul, MS	24	5,536	3	0	1	0	0.0193	0.0024–0.0530
Cristalina, GO	104	15,844	49	17	2	1	0.0071	0.0015–0.0170
Londrina, PR	97	20,512	1	0	1	0	0.0051	0.0006–0.0140
Ponta Grossa, PR	23	4,384	1	0	1	0	0.0200	0.0024–0.0551
Tasso Fragoso, BA	67	7,640	1	0	1	0	0.0072	0.0009–0.0201
Uberlândia, MG	132	28,165	10	2	3	1	0.0075	0.0020–0.0163
Bagé, RS	106	19,714	9	0	4	0	0.0116	0.0038–0.0236
Correntina, BA	64	15,040	0	0	0	0	0.0038	0.0001–0.0140
Luís Eduardo Magalhães, BA	94	23,200	25	4	2	1	0.0078	0.0016–0.0187
Chapadão do Sul, MS	63	15,232	2	0	1	0	0.0077	0.0009–0.0214
*Brazil (2019/20)*	*1587*	*315,748*	*149*	*41*	*26*	*7*	*0.0042*	*0.0028*–*0.0060*
**2020/21 soybean season**								
Campo Verde, MT	139	20,799	28	22	9	7	0.0177	0.0085–0.0301
Cristalina, GO	85	19,040	11	3	3	2	0.0115	0.0031–0.0251
Sapezal, MT	56	9,216	0	0	0	0	0.0043	0.0001–0.0159
Correntina, BA	135	25,797	18	14	5	3	0.0109	0.0040–0.0212
Cascavel, PR	121	18,000	4	2	1	1	0.0041	0.0005–0.0113
Londrina, PR	93	18,080	0	0	0	0	0.0026	0.0001–0.0097
Maracaju, MS	99	17,433	17	0	4	0	0.0124	0.0040–0.0252
Uberlândia, MG	101	20,717	0	0	0	0	0.0024	0.0001–0.0089
Rio Verde, GO	120	18,057	2	0	1	0	0.0041	0.0005–0.0114
Passo Fundo, RS	65	9,051	0	0	0	0	0.0037	0.0001–0.0138
Campo Grande, MS	87	15,776	0	0	0	0	0.0028	0.0001–0.0104
Roda Velha, BA	102	15,445	0	0	0	0	0.0024	0.0001–0.0089
Campo Mourão, PR	128	23,562	48	29	9	8	0.0192	0.0092–0.0326
Tasso Fragoso, MA	64	10,236	0	0	0	0	0.0038	0.0001–0.0140
Lucas do Rio Verde, MT	68	11,424	0	0	0	0	0.0036	0.0001–0.0132
Conchal, SP	50	8,928	0	0	0	0	0.0048	0.0001–0.0177
*Brazil (2020/21)*	*1513*	*261,561*	*128*	*70*	*32*	*21*	*0.0054*	*0.0037–0.0074*

The research did not involve the collection of plant material in nature. All plants used in the study were grown from commercially available seeds. The study complies with relevant institutional, national, and international guidelines and legislation.

#### Phenotypic resistance monitoring of *C. includens* using a diagnostic concentration of Cry1Ac protein

Diet-incorporated bioassays were performed with a Cry1Ac formulated product (MVP II, *Pseudomonas* encapsulated Cry1Ac from Dow Chemicals, San Diego, CA, containing 11.14% of active Cry1Ac protein). As described in Yano et al.^10^, 5.6 µg of Cry1Ac protein/mL of diet was used as the diagnostic concentration for resistance monitoring in *C. includens* populations (SI Table 1). For the bioassays, the Cry1Ac protein was diluted in distilled water to 56 µg/mL and 4 mL of this solution was poured into a 50-mL Falcon tube. The tube was then filled with 36 mL of artificial diet^28^. The mixture was homogenized in a vortex mixer for $\sim$ 40 s. Then, 1 mL of diet containing Cry1Ac protein was poured into each well of a 128-well bioassay tray (BIO-BA-128; CD International Inc., Pitman, NJ). After the diet had dried, one neonate larva (< 24 h old) from the F_1_ generation was placed into each well with a fine brush. Trays were sealed with a plastic adhesive (BIO-CV-16; CD International Inc.) that allowed air exchange and kept in a climatic chamber at 25 ± 2 °C, 70 ± 10% RH and 14:10 h (L:D) photoperiod. Mortality was recorded at 7 days. A total of 1,024 neonates/population were tested. Mortality data for each population were plotted on a time scale from 2015/16 (2016) to 2020/21 (2021). Data from 2009/10–2014/15 were previously reported in Yano et al.^10^ and plotted herein.

#### Genotypic Cry1Ac resistance monitoring of *C. includens* using F_2_ screens

To estimate the Cry1Ac resistance allele frequency in Brazilian populations of *C. includens*, we utilized the F_2_ screen method proposed by Andow and Alstad^29^. From 2016/17 (2017) to 2020/21 (2021), a total of 74 populations of *C. includens* were screened (SI Table 2). The collected larvae were transported to a laboratory and kept on artificial diet^28^. After pupation, pupae were separated by sex and used to establish multiple single-pair mating couples under laboratory conditions. The offspring (F_1_ progeny) of each single-pair mating (isoline) were reared in artificial diet, and pupae were transferred to a PVC cylindrical cage (20 cm height × 10 cm diameter) lined with paper (oviposition substrate) and covered with a mesh fabric until adult emergence. Adults were fed with a 10% honey solution provided on cotton inside a plastic cup. Eggs were collected and kept in plastic cups with filter paper moistened with water. F_2_ generation neonates (≤ 24 h old) were then used for screening. For the bioassays, leaves from the upper third of greenhouse-grown soybean plants were collected and kept in a refrigerator until use. Each isoline was tested in 128-well bioassay trays (BIO-BA-128; CD International Inc.) containing a 2% agar solution and one soybean leaf disc of 1.7 cm diameter. Two neonate larvae (< 24 h old) were placed in each well with a fine brush, with a target number of 128 neonates tested/isoline in 2016/17 (2017) and 256 neonates tested/isoline for 2017/18 to 2020/21 (2018–2021). Then, plates were sealed and placed under the same environmental conditions described above. Survivorship was recorded after 4 days. If any survivors were found on MON 87701 × MON 89788 soybean after 4 days, the leftover leaf discs were tested for Cry1Ac expression using QuickStix kits for Cry1Ac (Envirologix, Portland, Maine, USA). An isoline was considered positive (putative resistant) if any survivor was detected on MON 87701 × MON 89788 soybean. To estimate the resistance allele frequency, we used the equation presented in Andow and Alstad^29^, and the 95% confidence intervals were estimated as described by Andow and Alstad^30^. The resistance allele frequency was calculated using the function *binom.bayes* from the package *binom* in R statistical software—R version 4.0.2^31^.

**Table 2:** Concentration-mortality response (ng Cry1Ac/cm^2^) of *C. includens* and *R. nu* neonates exposed to purified Cry1Ac protein in diet-overlay bioassay.

Species	*n*	Fit of probit lines			LC_50_ (95% CI)^‡^	TR^§^
		Slope ± SE	χ2 (*df*^†^)	*P*		
*R. nu*	399	1.60 ± 1.49	1.77 (5)	0.22	> 74,600.00 (not estimated)	> 2,709
*C. includens*	192	2.16 ± 0.48	7.95 (3)	0.16	27.53 (15.23–43.62)	-

^†^*df* = degrees of freedom.

^‡^LD_50_ and 95% confidence interval (95% CI).

^§^Tolerance Ratio (TR) = LC_50_ of *R. nu*/LC_50_ of *C. includens*.

### Reactive resistance monitoring: investigating unexpected injury to MON 87701 × MON 89788 soybean in Brazil

After reports of unexpected injury to MON 87701 × MON 89788 soybean commercial fields in Brazil, an investigation was initiated. Larvae were sampled from these fields to identify the lepidopteran species attacking the plants. Identifications of lepidopteran species were based on Herzog^32^, Navarro et al.^33^ and Gilligan and Passoa^34^ and indicated that *R. nu* and *C. aporema* were the species injuring MON 87701 × MON 89788 soybeans. These species were then investigated for resistance to Cry1Ac as described below.

#### Testing sampled *R. nu* field populations

To understand the unexpected plant injury and survival of *R. nu* in MON 87701 × MON 89788 soybean fields in Brazil during the 2020/21 season, bioassays with Cry1Ac protein and leaf discs of MON 87701 × MON 89788 soybean were carried out. Diet-overlay bioassays using Cry1Ac protein were used to test a *R. nu* population collected in Paranapanema, SP, Brazil (SI Table 3). A susceptible *R. nu* laboratory colony was not available, so a susceptible laboratory population of *C. includens* was used as a standard for comparison of tolerance levels between the two species. The purified Cry1Ac insecticidal protein was produced by Bayer Crop Science US (Chesterfield, MO, USA). Proteins were isolated from fermentation broths of recombinant *B. thuringiensis* strains transformed to express individual toxins, like described in Chen et al.^35^ Bioassays were performed in 96-well bioassay trays with 200 μL of artificial diet per well. Seven concentrations of Cry1Ac were prepared by dilution in TX buffer (0.005% Triton X-100, 10 mM Tris–HCl, pH 7.4). The control treatment was composed of TX buffer. After preparation, 20-µl protein samples were overlaid on the diet surface of each well and ventilated until the excess moisture dissipated. After drying, each well was infested with a single neonate larva (< 24 h old) using a fine brush. Mortality was recorded at 6 days. Lethal concentrations (LC_50_) and 95% confidence intervals were estimated using probit analysis in SAS 9.1^36^.

**Table 3:** Percent mortality (95% CIs) of *R. nu* populations resistant to MON 87701 × MON 89788 soybean in complementation test for allelism.

Cross	*n*	MON 87701 × MON 89788 soybean^†^	Non-*Bt* soybean^†^	Complementation
Paranapanema 2020 × Uberaba 2020	192	4.6 (2.3–8.5) a	4.6 (2.3–8.5) a	Yes
Paranapanema 2020 × Taquarituba 2020	192	10.9 (7.1–16.0) a	7.8 (4.6–12.3) a	Yes
Paranapanema 2020 × Taquarituba 2021	192	3.6 (1.6–7.1) a	2.6 (1.0–5.8) a	Yes
Paranapanema 2020 × Perdizes 2021	192	7.8 (4.6–12.3) a	7.8 (4.6–12.3) a	Yes

^†^Values represent means (95% CI). Mortality on MON 87701 × MON 89788 soybean and non-*Bt* soybean followed by the same letter in each row are not significantly different due to overlap of 95% CIs. No differences on mortality among MON 87701 × MON 89788 soybean and non-*B*t soybean indicates that the resistance alleles are probably in a same locus.

Leaf-disc bioassays were also performed with *C. includens* and *R. nu* field populations sampled from Brazil and Argentina (SI Table 3). Bioassays were conducted in the respective country of sampling with neonates from the F_1_ to F_2_ generations in Argentina and from the F_1_ generation in Brazil. The MON 87701 × MON 89788 soybean and non-*Bt* soybean were grown in a greenhouse. Seeds were sown on the ground within a greenhouse in Brazil, whereas in Argentina they were cultivated in 5 L plastic pots with 1 plant/pot. Nutrients and water were provided according to the necessity during plant development. Bioassays were performed with leaves of V6- to R3-growth-stage plants^37^ following the leaf-disc bioassay method previously described, but with the difference that a single neonate (< 24 h old) was placed in each well. A total of 128 neonates was tested for each population/treatment combination. Bioassay were carried out with 8 replicates of 16 larvae in Brazil and 4 replicates of 32 larvae in Argentina. Mortality was recorded at 4 days counting the number of dead larvae in each replicate. The percent mortality on MON 87701 × MON 89788 soybean and non-*Bt* soybean. The total number of tested and dead insects on MON 87701 × MON 89788 soybean and non-*Bt* soybean were used to estimate the 95% confidence interval (CI) for the probability of mortality, according to binomial distribution. The statistical analysis of the data from these bioassays was made using the function *binom.probit* from the package *binom* in R statistical software—R version 4.0.2^31^. Percent mortality on MON 87701 × MON 89788 soybean and non-*Bt* soybean were considered significantly different when their 95% CI did not overlap.

To determine whether the same resistance allele was present in populations of *R. nu* sampled from distinct locations in Brazil, we also ran a complementation test for allelism. For this, the field population of *R. nu* from Paranapanema sampled in 2020 was crossed with four other field populations (Uberaba 2020, Taquarituba 2020, Taquarituba 2021, Perdizes 2021) (SI Table 3). At least 15 pairs were used for each crossing. The F_1_ progeny were tested using leaf-disc bioassays as previously described, with 12 replicates of 16 larvae/cross, totaling 192 neonates tested on MON 87701 × MON 89788 soybean and on non-*Bt* soybean. Mortality was recorded after 4 days, and data were compared as earlier described.

#### Testing sampled *C. aporema* field populations

To understand the unexpected injury and survival of *C. aporema* on MON 87701 × MON 89788 soybean fields in Brazil during the 2020/21 soybean season, four populations were collected in different MON 87701 × MON 89788 soybean fields with unexpected injury (SI Table 3). Neonates from these populations (F_1_ generation) were used in leaf-disc bioassays of MON 87701 × MON 89788 soybean and non-*Bt* soybean as described in the previous section. Because only a limited number of eggs were available from the mass mating of field insects, only 13 neonates from the Itararé population and 32 neonates from the Cristalina, Perdizes and Tibagi populations were tested in each treatment. Mortality was recorded at 4 days. The mortality data of each population on MON 87701 × MON 89788 soybean and non-*Bt* soybean were compared as earlier described.

#### Abundance of lepidopteran pests on commercial fields of MON 87701 × MON 89788 soybean and non-*Bt* soybean in Brazil

Lepidopteran larvae were sampled from 395 geographically distinct soybean fields during the 2020/21 cropping season. Each location comprised a non-*Bt* (Roundup Ready [RR]) soybean field and a MON 87701 × MON 89788 soybean (Intacta RR2 PRO) field. A beat cloth (1-m length) was used to sample larvae. A total of 10 beats in a zig-zag pattern was considered the sampling unit. To minimize border effects, sampling was initiated from at least 20 m from the border of soybean fields in the Southern region farms, where farms are smaller (average size of farms less than 100 ha), and 100 m in the Northeast and Central-West regions, where larger farms are predominant (average size of farms greater than 150 ha). For each location, beat-cloth sampling started in the non-*Bt* field; when at least 1 lepidopteran larva per meter was found, samples were also taken from a nearby MON 87701 × MON 89788 soybean field at a similar plant growth stage. The presence of Cry1Ac protein in MON 87701 × MON 89788 soybean plants and the absence of this protein in non-*Bt* soybean plants were checked for Cry1Ac expression in all fields using QuickStix kits for Cry1Ac (Envirologix). Sampled larvae from each beat-cloth were transferred to a labeled 50-mL conical Falcon tube containing propylene glycol. Tubes containing larvae were then sent to the laboratory and kept in a freezer (− 20 °C) until identification. Identification of lepidopteran larvae were based on Herzog^32^, Sosa-Gómez et al.^38^, Navarro et al.^33^ and Gilligan and Passoa^34^. Sampling fields were grouped according to the location within Embrapa’s (Embrapa Soybean) soybean variety regionalization named “edaphoclimatic regions” and “soybean macroregions”^39^.

Statistical modeling was used to characterize soybean geographic variation in pest abundance in non-*Bt* soybean and MON 87701 × MON 89788 soybean. Specifically, random effects for edaphoclimatic regions were estimated separately for each pest and field type (non-*Bt* soybean and MON 87701 × MON 89788 soybean) using a linear mixed-effects model for larval count data with Poisson link, with edaphoclimatic region and soybean macroregion treated as random effects, and edaphoclimatic region nested within soybean macroregion. An advantage of random effects estimates, compared to fixed effects estimates or simply using average counts by region, is that they properly account and adjust for sample size differences between regions, providing more accurate predictions of true pest density. The random effects estimates of pest abundance by edaphoclimatic region were summarized using choropleth maps. Analyses were performed with R statistical software—R version 4.0.2^31^ using the *glmer* function in R package “lmer”.

#### Refuge compliance and MON 87701 × MON 89788 soybean adoption in Brazil

Data on MON 87701 × MON 89788 soybean adoption and strict refuge compliance across 46 to 51 mesoregions (number varied among years) for the 2014/15 to 2019/20 cropping seasons were obtained from market research companies Kynetec (2014/15–2017/18) and Spark (2018/19–2019/20). Mesoregions are Brazilian geographical division in a federative unit (state) defined by social process, natural and network of communication and places^40^. Figure 1 of the Supporting Information (SI) shows the mesoregions used in the analysis. A linear regression analysis was performed with the percentage of refuge compliance as a function of MON 87701 × MON 89788 adoption, using GraphPad Prism 8—version 8.1.2 (GraphPad Software, San Diego, CA, USA)^41^.

**Figure 1:**
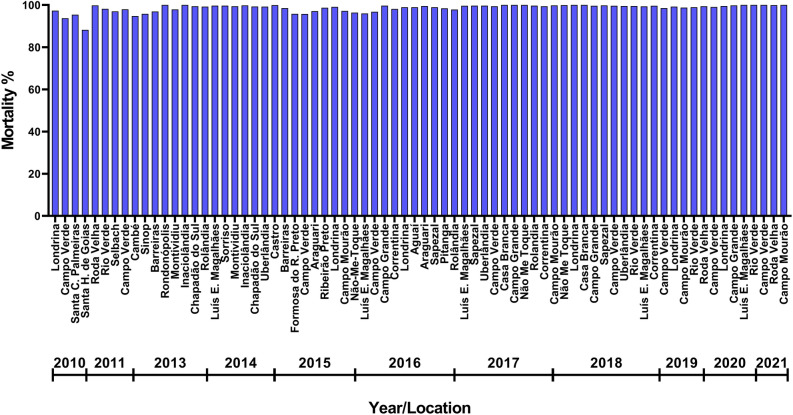
Susceptibility monitoring of *C. includens* populations from Brazil to Cry1Ac protein during 2009/10–2020/21 using diagnostic concentration bioassay. Data from 2009/10 to 2014/15 were reported previously in Yano et al.^10^.

## Results

### Proactive MON 87701 × MON 89788 soybean resistance monitoring of ***C. includens***

#### Phenotypic resistance monitoring in *C. includens* using a diagnostic concentration of Cry1Ac protein

There was high mortality in all field populations of *C. includens* collected from 2015/16 to 2020/21 (42 field populations) when exposed to the Cry1Ac diagnostic concentration of 5.6 µg of Cry1Ac protein per mL of diet (Fig. 1). The neonate mortality in this period ranged from 96 to 100%, not differing over time. Compared with data from 2009/10–2014/15, there was also no obvious change in the susceptibility to Cry1Ac protein in populations of *C. includens* from Brazil over the evaluated years (Fig. 1).

#### Genotypic Cry1Ac resistance monitoring in *C. includens* using F_2_ screens

**Figure 2:**
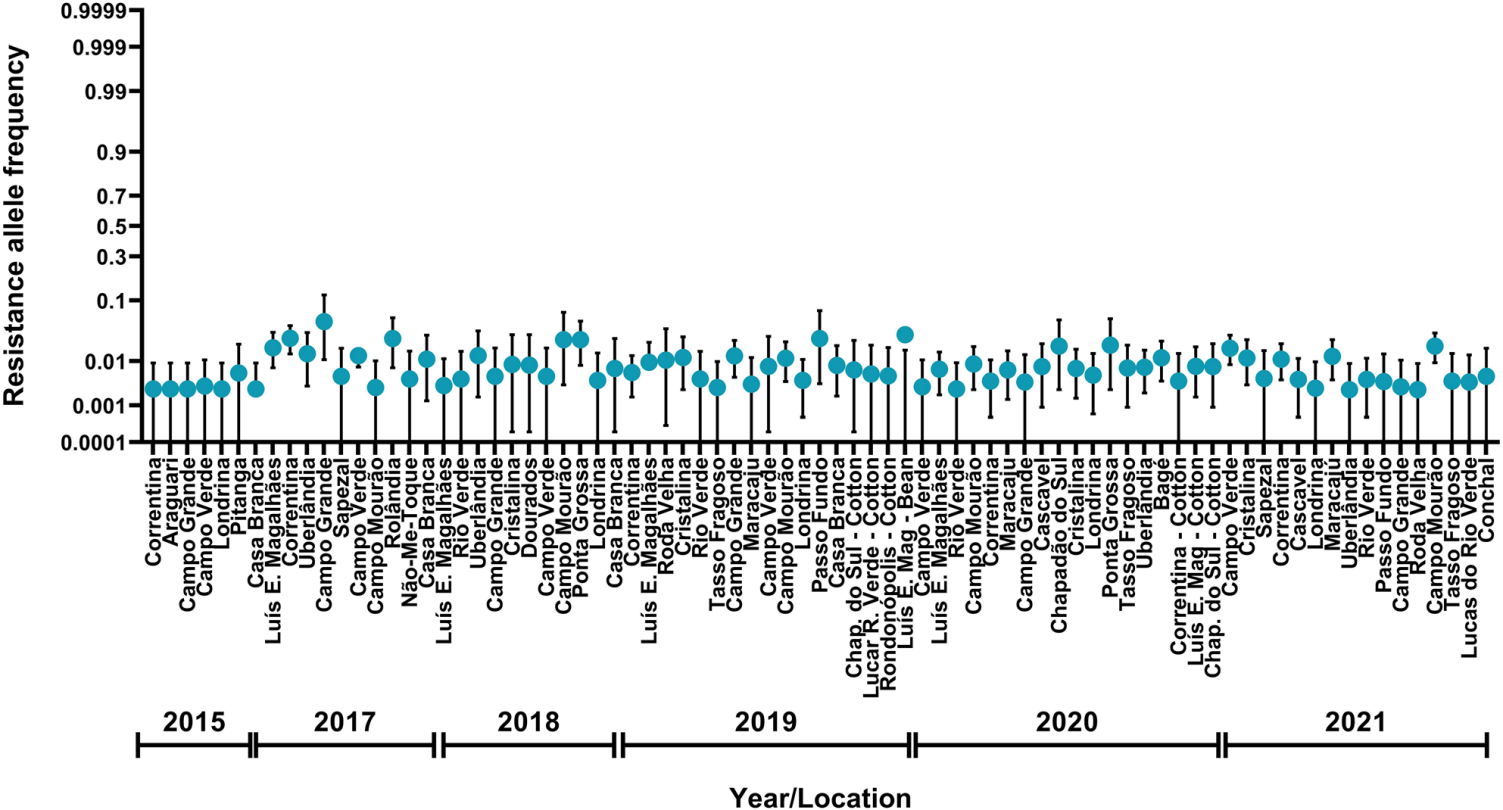
Frequency of resistance alleles conferring resistance of *C. includens* to MON 87701 × MON 89788 soybean in Brazil. Data from 2014/15 were previously reported by Yano et al.^10^.

From 2016/17 to 2020/21, a total of 74 populations, 5,436 isolines and more than one million neonates of *C. includens* were screened on leaf discs of MON 87701 × MON 89788 soybean. Resistance alleles were detected in 119 isolines (considered positive when any neonate survived on MON 87701 × MON 89788 soybean at 4 days), with 61 isolines presenting larvae that reached pupal stage while feeding on MON 87701 × MON 89788 soybean leaves (Table 1). The estimated resistance allele frequency decreased from 0.0115 (95% CI, 0.0076–0.0162) in 2016/17 to 0.0033 (95% CI, 0.0013–0.0062) in the 2017/18 soybean season. However, resistance allele frequency remained similar in subsequent seasons: 0.0061 (95% CI, 0.0041–0.0084) in 2018/19, 0.0042 (95% CI, 0.0028–0.0060) in 2019/20, and 0.0054 (95%, 0.0037–0.0074) in 2020/21. Across crop seasons and regions, the frequency of resistance alleles to MON 87701 × MON 89788 soybean was also stable in *C. includens* populations from 2016/17 to 2020/21, suggesting no relevant shifts in susceptibility (values distributed among 0.0024 to 0.05) (Fig. 2), confirming the highly efficacious control of this key pest by MON 87701 × MON 89788 soybean.

### Reactive resistance monitoring: investigating unexpected injury to MON 87701 × MON 89788 soybean caused by ***R. nu*** and ***C. aporema*** in Brazil

#### Testing sampled *R. nu* field populations

The Cry1Ac concentrations used to estimate LC_50_ in *R. nu* from a MON 87701 × MON 89788 soybean field in Brazil ranged from 1,165 to 74,600 ng/cm^2^. At these concentrations, the mortality of the *R. nu* population ranged from 8.3 to 25.4%. The LC_50_ of Cry1Ac and respective 95% CI for this population could not be accurately estimated and was considered higher than 74,600 ng/cm^2^ (maximum concentration tested) (Table 2). In contrast, the susceptible *C. includens* laboratory population showed 100% mortality at 1.165 ng/cm^2^, with an LC_50_ of Cry1Ac of 27.53 (95% CI 15.23–43.62) ng/cm^2^ (Table 2). The estimated ratio of tolerance to Cry1Ac protein was > 2,709 for the *R. nu* population tested. Significant differences in susceptibility to Cry1Ac protein between species were also verified by the equality (χ^2^ = 230.0; *df* = 2; *P* < 0.001) and parallelism (χ^2^ = 130.3; *df* = 1; *P* < 0.001) tests, which indicated that the mortality curves had distinct parameters (intercepts and slopes).

**Figure 3:**
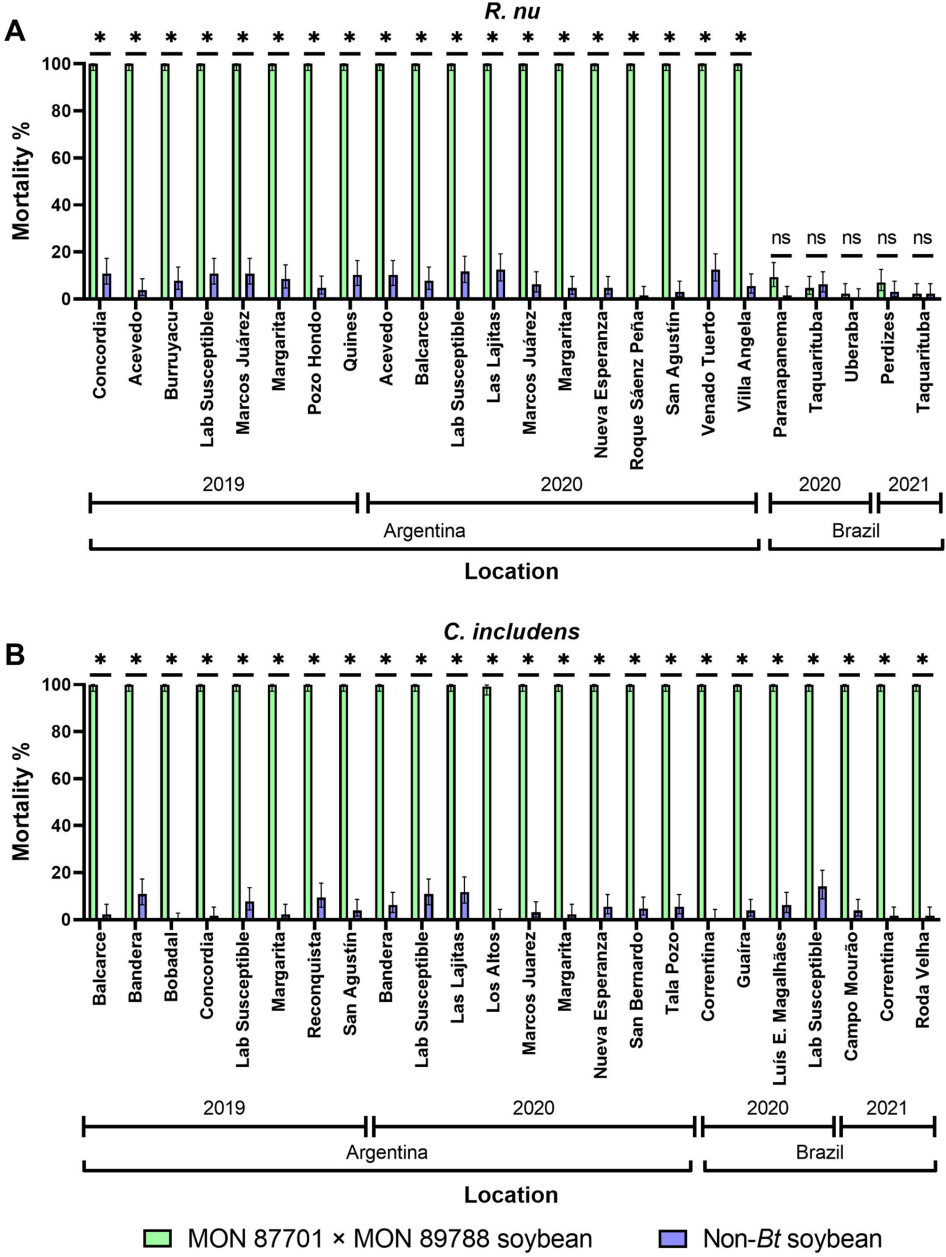
Mortality of *R. nu* (**A**) and *C. includens* (**B**) populations sampled from soybean fields in Argentina and Brazil on MON 87701 × MON 89788 soybean and non-*Bt* soybean. The asterisk (*) indicated that the mortality on MON 87701 × MON 89788 soybean and non-*Bt* soybean differed significantly due to non-overlap of 95% CIs. ns = non-significant.

When neonates of the *R. nu* population tested above and four other populations obtained in MON 87701 × MON 89788 soybean fields in Brazil were exposed to MON 87701 × MON 89788 soybean leaf discs, there was no higher than 9.4% mortality, not differing significantly from those verified on non-*Bt* soybean controls (Fig. 3A). In contrast, *R. nu* populations from Argentina presented 100% mortality on MON 87701 × MON 89788 soybean leaf discs and no higher than 12.5% mortality on non-*Bt* soybean. Similar results to those for *R. nu* from Argentina were obtained for *C. includens* populations from Brazil and Argentina exposed to MON 87701 × MON 89788 soybean (> 99.2% mortality) and non-*Bt* soybean (< 15% mortality) (Fig. 3B).

In the complementation study, there were no significant differences in the mortality of progeny from four crosses involving five populations of *R. nu* from distinct MON 87701 × MON 89788 soybean fields in Brazil when exposed to MON 87701 × MON 89788 soybean (3.6 to 10.9%) and non-*Bt* soybean (2.6 to 7.8%) (Table 3). These results provide evidence against the hypothesis that the resistance alleles in any of the populations were at different loci and therefore suggest that the resistance alleles are probably at the same locus.

Overall, our results suggest a genetically based decrease in susceptibility to Cry1Ac protein expressed in soybean in the Brazilian populations of *R. nu* tested.

#### Testing sampled *C. aporema* field populations

**Figure 4:**
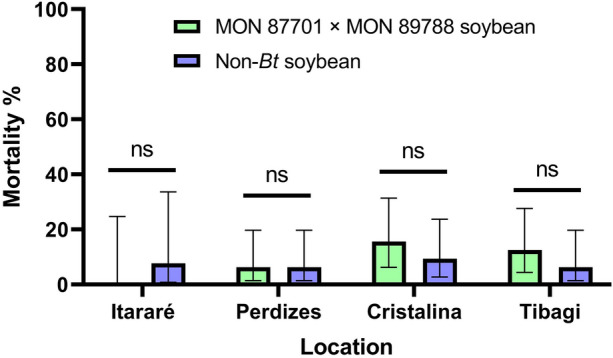
Percent mortality (95% CI) of *C. aporema* populations on MON 87701 × MON 89788 soybean and non-*Bt* soybean. ns = non-significant.

The *C. aporema* populations sampled from Cristalina, Itararé, Perdizes and Tibagi showed similar mortality on MON 87701 × MON 89788 soybean (0.0 to 15.6%) and non-*Bt* soybean (6.3 to 9.3%) (Fig. 4). These results indicated a decrease in susceptibility to Cry1Ac protein expressed in MON 87701 × MON 89788 soybean by *C. aporema* populations from Brazil.

#### Abundance of lepidopteran pests on commercial fields of MON 87701 × MON 89788 soybean and non-*Bt* soybean in Brazil

**Figure 5:**
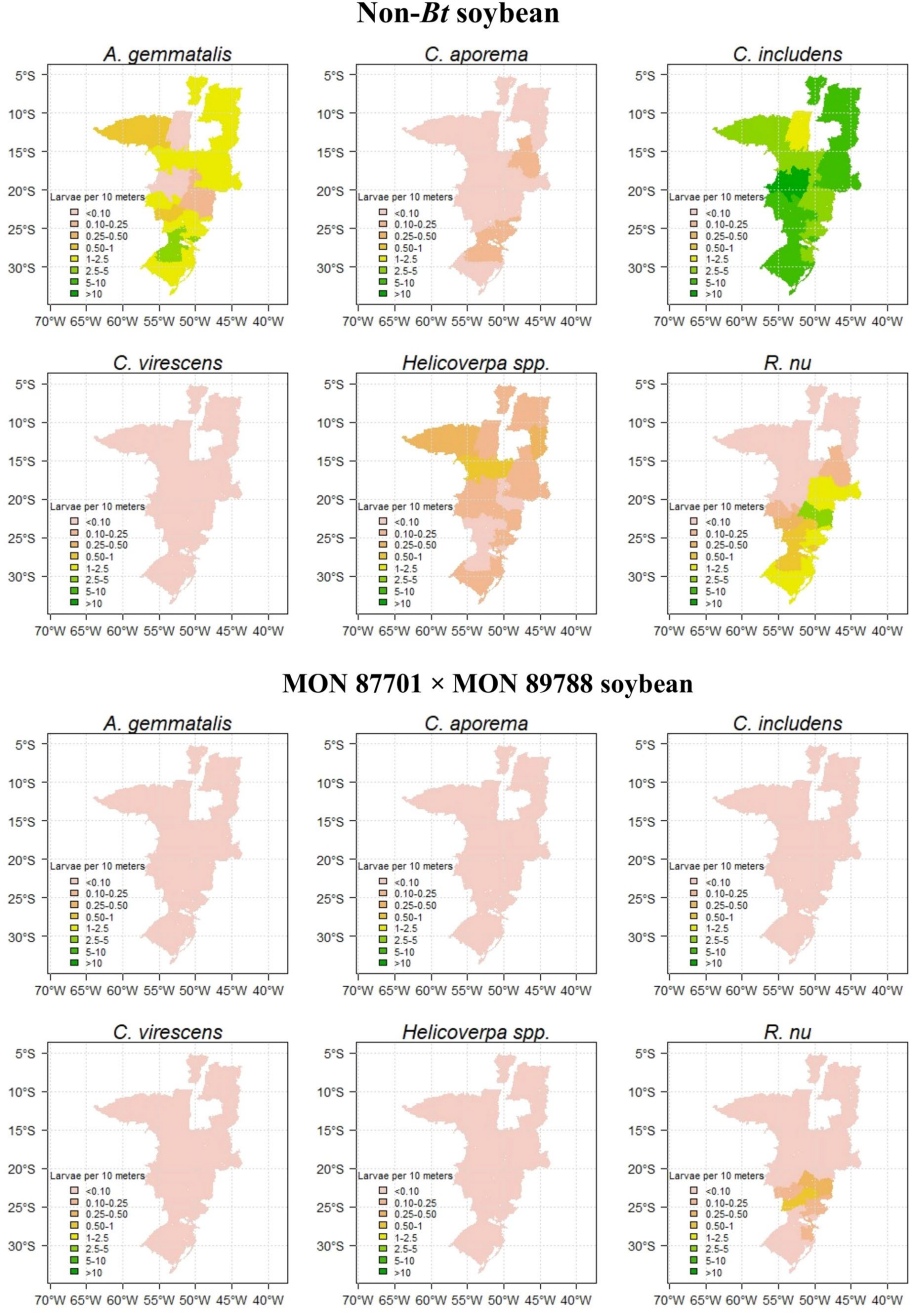
Incidence of lepidopteran pests in MON 87701 × MON 89788 soybean and non-*Bt* soybean in Brazilian fields sampled during the 2020/21 cropping season. A random effects statistical model was used to estimate pest abundance for each edaphoclimatic region. Separate analyses were conducted for each pest and field type. Pest abundance estimates were summarized using choropleth maps, where each edaphoclimatic region is color-coded according to its estimated pest abundance (different colors are assigned for estimates in the ranges < 0.10, 0.1–0.25, 0.25–0.5, 0.5–1, 1–2.5, 2.5–5, 5–10, and > 10 larvae/10 m). In all maps that show more than one color, there is statistically significant variation across edaphoclimatic regions (p < 0.05). Maps were generated using R statistical software—R version 4.0.2 (https://www.R-project.org/).

A random effects statistical model was used to estimate pest abundance for each edaphoclimatic region. Separate analyses were conducted for each pest and field type. Pest abundance estimates were summarized using choropleth maps, where each edaphoclimatic region is color-coded according to its estimated pest abundance (different colors are assigned for estimates in the ranges < 0.10, 0.1–0.25, 0.25–0.5, 0.5–1, 1–2.5, 2.5–5, 5–10, and > 10 larvae/10 m). In all maps that show more than one color, there is statistically significant variation across edaphoclimatic regions (*P* < 0.05) (Fig. 5).

The visualization of geographic variation in lepidopteran pest abundance in MON 87701 × MON 89788 soybean and non-*Bt* soybean fields shows that *C. includens* was present at high density in 301 of the 395 sampling locations in non-*Bt* soybean (76.2% of fields) but nearly absent (1.01%) from MON 87701 × MON 89788 soybean fields (Fig. 5). *Anticarsia gemmatalis, Helicoverpa* spp. and *C. virescens* were also nearly absent (< 0.51%) from MON 87701 × MON 89788 soybean in almost all samples but were detected in 25.3, 11.1 and 2.3% of the non-*Bt* soybean fields, respectively (Fig. 5). Among the species analyzed, *A. gemmatalis* had the second-highest density on non-*Bt* soybean across the regions examined (Fig. 5). *Crocidosema aporema* larvae was observed at low density in non-*Bt* soybean fields in some areas of south and central Brazil (Fig. 5); however, the beat-cloth method is not the best method to sample this species so its incidence might have been underestimated. In contrast to other species, *R. nu* larvae appeared in a moderate number of non-*Bt* soybean fields from south to central Brazil. Its occurrence was also detected in 5.5% of the MON 87701 × MON 89788 soybean fields sampled, mainly in growing areas located in Paraná and São Paulo states (Fig. 5).

### Refuge compliance and MON 87701 × MON 89788 soybean adoption in Brazil

**Figure 6:**
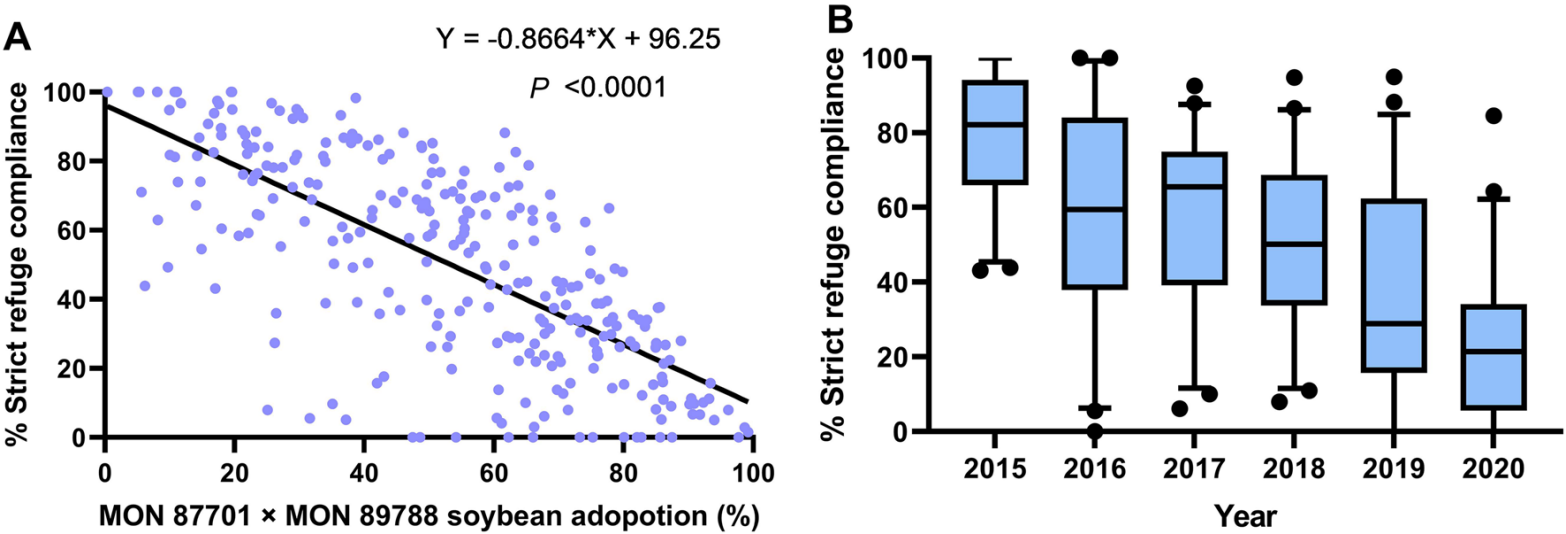
Relationship between adherence to structured refuge requirements and the adoption of MON 87701 × MON 89788 soybean across mesoregions (**A**) and crop seasons (**B**) in Brazil. Data on MON 87701 × MON 89788 soybean adoption and strict refuge compliance across mesoregions for the 2014/15 to 2019/20 cropping seasons were obtained from market research companies Kynetec (2014/15–2017/18) and Spark (2018/19–2019/20). The middle horizontal line within each box in (**B**) represents the median; the bottom and top edges of the boxes represent the 25th and 75th percentiles, respectively.

The compliance to refuge requirements significantly decreased with increasing MON 87701 × MON 89788 adoption over mesoregions (F = 290.8, df = 1,281, R^2^ = 0.51, *P* < 0.0001) (Fig. 6A). The median refuge compliance in 2014/15 was 82.1% and dropped to 21.4% in 2019/20 (Fig. 6B).

## Discussion

The proactive phenotypic and genotypic monitoring of Cry1Ac resistance did not show shifts in the susceptibility of C*. includens* that would lead to unexpected injury on MON 87701 × MON 89788 soybean in the field*.* Although an initial shift was observed from the genotypic resistance baseline monitoring carried out in 2014/15^10^, our findings indicated that the resistance allele frequency did not increase significantly over the following years. Even though some *C. includens* larvae tested in our F_2_ screening survived on MON 87701 × MON 89788 soybean leaves, most of the larvae that developed into adults did not produce fertile eggs, suggesting an incomplete resistance to Cry1Ac in *C. includens*. If a major recessive trait confers resistance, 1/16 of the F_2_ larvae are expected to be homozygous resistant and therefore able to complete their life cycle on a *Bt* plant^29,42^. Because the percentage of resistant larvae was much smaller than 1/16, we hypothesized that minor genes were responsible for the survival of *C. includens* on MON 87701 × MON 89788 soybean in our F_2_ screening. The F_2_ screen method proposed by Andow and Alstad^29^ might not be the best way to estimate the resistance allele frequency of minor alleles involved with resistance, since it was developed to estimate the frequency of major resistance alleles. Although the resistance allele frequency might be biased with that method, it is still a good estimation of resistance allele frequency over the time, as we are using the same method over years. So far, no unexpected injury on MON 87701 × MON 89788 soybean was reported by *C. includens*, confirming the low resistance allele frequency detected in the F_2_ screens. We further hypothesized that the resistance alleles detected in our genetic monitoring might be strongly associated with fitness, since the *C. includens* adults did not produce viable offspring.

The high-dose/refuge strategy used for management of resistance to MON 87701 × MON 89788 soybean seems to have contributed to maintaining a low frequency of resistance in natural populations of *C. includens*. In addition to the results previously published by MacRae et al.^1^ and Bernardi et al.^4^, further evidence supporting the functional recessiveness of Cry1Ac resistance in *C. includens* exposed to MON 87701 × MON 89788 soybean was obtained when a laboratory strain of *C. includens* selected on Cry1Ac *Bt* cotton was crossed with susceptible insects and the F_1_ progeny showed complete mortality on MON 87701 × MON 89788 soybean leaves^43^.

In contrast to previous results, unexpected injury caused by secondary target pests *R. nu* and *C. aporema* was detected in MON 87701 × MON 89788 soybean fields during the 2020/21 season. Although susceptible reference populations of these species were not available, differences were detected in the susceptibility to MON 87701 × MON 89788 soybean among *R. nu* populations from Brazil and Argentina. Diet-overlay bioassays using Cry1Ac protein also indicated a high tolerance ratio (> 2,709-fold) for *R. nu* sampled from MON 87701 × MON 89788 soybean fields compared to *C. includens* from non-*Bt* soybean. A study conducted before the commercial launch of MON 87701 × MON 89788 soybean showed that the LC_50_ of Cry1Ac was 1.53 and 0.70 µg/ml of artificial diet for *C. includens* and *R. nu*, respectively^44^. These results indicated a tolerance ratio of *R. nu* in relation to *C. includens* of 0.45-fold, evidencing that these Plusiinae had similar susceptibility to Cry1Ac in Brazil. The complementation test results suggested that the resistance alleles are probably at the same locus in different field populations of *R. nu* sampled from MON 87701 × MON 89788 soybean fields in Brazil. Similarly, offspring of *C. aporema* sampled from MON 87701 × MON 89788 soybean fields presented similar mortality on *Bt* and non-*Bt* soybean, suggesting a genetically based decrease in the susceptibility to Cry1Ac protein.

The development of resistance of *R. nu* and *C. aporema* to MON 87701 × MON 89788 soybean may have also been influenced by the biological characteristics of these species and of the cropping systems in Brazil. Historically, *R. nu* has been a soybean pest in the southern part of South America (Rio Grande do Sul state in Brazil, Uruguay and Argentina)^24,26,45^. In a prior field survey in 2019 and 2020, *R. nu* occurred at a low level in Brazil^7^. However, *R. nu* has increased in abundance in soybean areas at lower latitudes, which may indicate an adaptation of *R. nu* populations to warmer climate conditions before evolving resistance to MON 87701 × MON 89788 soybean. Low genetic distances between populations from different South American countries also suggest the absence of geographical isolation amongst these natural populations^45^. For *C. aporema*, the short lifecycle (12 days) allows the development of several generations per season, therefore increasing the selection pressure for resistance. The planting of common bean (*Phaseolus vulgaris* L.) combined with the presence of volunteer MON 87701 × MON 89788 soybeans after crop harvest in field areas where these pests were found in southern Brazil creates a “green bridge” during the soybean off-season and increases the sequential exposure of these pests to MON 87701 × MON 89788 soybean. Furthermore, the low adoption of structured refuges together with the overuse of insecticides in non-*Bt* areas^46^ likely played an important role in establishing field-evolved Cry1Ac resistance in these species. Reductions in refuge compliance might compromise the high-dose/refuge strategy recommended for MON 87701 × MON 89788 soybean in Brazil. The common understanding is that the high yield of MON 87701 × MON 89788 soybean varieties relative to most of the commercially available non-*Bt* soybean varieties and the relative ease of pest management relative to non-*Bt* soybean have led some growers to not comply with the refuge recommendation in Brazil. In addition, the planting of refuges is not mandatory in Brazil, and recommendations are mainly promoted by technology providers, adding more challenges for compliance to IRM requirements^47^. According to Tabashnik and Carrière^11^, low refuge compliance was a key factor in cases of field-evolved resistance to *Bt* plants worldwide. Moreover, the lack of adoption of Integrated Pest Management (IPM) practices, particularly in refuge areas, can increase the spraying of foliar insecticides in these areas, thus reducing the effectiveness of this important IRM strategy and favoring pest resurgence by likely reducing the abundance of biological control agents^46^. Despite the characteristics of *R. nu* and *C. aporema* that might have favored resistance development, the question remains why Cry1Ac resistance to MON 87701 × MON 89788 soybeans evolved in these species but not in *C. includens,* the most abundant lepidopteran soybean pest in Brazil^7^, under the same refuge adoption and management settings as these other pests. The absence of field-evolved Cry1Ac resistance in *C. includens* in Brazil suggests that the mechanisms of Cry1Ac resistance in *C. includens* may be more complex and detrimental to fitness than those in *R. nu* (or *C. aporema*). Understanding the genes and molecular mechanisms involved in Cry1Ac resistance in *C. includens*, *R. nu* and *C. aporema* can provide important information to support additional modeling of the projected durability of MON 87701 × MON 89788 soybeans in Brazil.

General assessment of injury in soybean fields indicated that MON 87701 × MON 89788 soybean provides excellent protection against the main soybean lepidopteran pests in Brazil. Our sampling performed during the 2020/21 season showed that *C. includens*, *A. gemmatalis* and *Helicoverpa* spp. were nearly absent from MON 87701 × MON 89788 soybean fields, showing the consistent efficacy of MON 87701 × MON 89788 soybean in managing these lepidopteran species. Our results also demonstrate that *C. includens* and *A. gemmatalis* remain the most abundant primary lepidopteran pests in non-*Bt* soybean fields, confirming the results from previous research^7^. These species were widely distributed across the soybean-growing regions of Brazil, whereas *R. nu*, *Helicoverpa* spp. and *C. aporema* were less prevalent. It is important to note, however, that beat-cloth sampling does not provide an accurate count of *C. aporema*, so its occurrence might have been underestimated. From 1980 to the 2000s, *A. gemmatalis* was the main lepidopteran pest of soybean in Brazil^48,49^. However, from the early 2000s, *C. includens* emerged as the key pest, which was likely influenced by the expansion of the soybean crop in Brazil^50^. In our results, *R. nu* and *C. aporema* were restricted to soybean fields in the Mid-South of Brazil, probably due to their better adaptation to subtropical and temperate climate of this region^25–27^, whereas *Helicoverpa* spp. was present in soybean fields in the Central regions of Brazil. *Chloridea virescens* was practically absent from both MON 87701 × MON 89788 soybean and non-*Bt* soybean fields.

Overall, we can conclude that MON 87701 × MON 89788 soybean remains effective against *A. gemmatalis*, *C. includens*, *Helicoverpa* spp. and *C. virescens* after eight years of commercial plantings in Brazil. However, we also report the first evidence of field-evolved resistance to MON 87701 × MON 89788 soybean in the secondary soybean pests *R. nu* and *C. aporema*. Our results indicate the MON 87701 × MON 89788 soybean continues to be a highly valuable and efficacious IPM tool that soybean growers in Brazil can rely on to manage the major pests *C. includens* and *A. gemmatalis* despite the localized cases of Cry1Ac resistance documented in secondary soybean lepidopteran pests. However, the increasing adoption of *Bt* soybean technology and the decrease in structured refuge compliance, along with these first and still localized cases of Cry1Ac resistance documented in secondary lepidopteran pests, highlights the importance of IRM practices.

To maintain the benefits of MON 87701 × MON 89788 soybean against primary target pests such as *C. includens* and *A. gemmatalis*, it is important to follow the refuge recommendation. The next generation of *Bt* soybean will continue to use Cry1Ac protein, now pyramided with Cry1A.105 and Cry2Ab2^51^. Therefore, the implementation of resistance management strategies is essential for the sustainability of both current and new *Bt* soybean varieties for managing lepidopteran pests in South America.

## Supplementary Information

Supplementary Information.
