# Supplementary material for: Resistance status of lepidopteran soybean pests following large-scale use of MON 87701 × MON 89788 soybean in Brazil
Source: Sci Rep. 2021 Oct 29;11:21323. doi: 10.1038/s41598-021-00770-0 (PMC8556339; doi:10.1038/s41598-021-00770-0)
Supplement: Supplementary file 1 — Supplementary Information. [file 41598_2021_770_MOESM1_ESM.docx]

Supporting Information (SI). Table 1. Populations of *C. includens* used in phenotypic resistance monitoring using Cry1Ac protein in diet-incorporated bioassays.

| Country | Species | Year | Host | State | Location | Latitude | Longitude |
| --- | --- | --- | --- | --- | --- | --- | --- |
| Brazil | *C. includens* | 2016 | Soybean | Bahia, BA | Luís Eduardo Magalhães | -12.1937421 | -46.0130690 |
| Brazil | *C. includens* | 2016 | Soybean | Mato Grosso, MT | Campo Verde | -15.3482768 | -54.9611493 |
| Brazil | *C. includens* | 2016 | Soybean | Mato Grosso do Sul, MS | Campo Grande | -20.7010000 | -54.8300000 |
| Brazil | *C. includens* | 2016 | Soybean | Bahia, BA | Correntina | -13.4802123 | -45.4089678 |
| Brazil | *C. includens* | 2016 | Soybean | Paraná, PR | Londrina | -23.5141795 | -51.1104167 |
| Brazil | *C. includens* | 2016 | Soybean | São Paulo, SP | Aguaí | -22.0500662 | -47.0402569 |
| Brazil | *C. includens* | 2016 | Soybean | Minas Gerais, MG | Araguari | -18.5771185 | -48.2221709 |
| Brazil | *C. includens* | 2016 | Soybean | Mato Grosso, MT | Sapezal | -13.1543688 | -58.6523480 |
| Brazil | *C. includens* | 2016 | Soybean | Paraná, PR | Pitanga | -24.6910934 | -51.7804143 |
| Brazil | *C. includens* | 2016 | Soybean | Paraná, PR | Rolândia | -23.2712800 | -51.4092050 |
| Brazil | *C. includens* | 2017 | Soybean | Bahia, BA | Luís Eduardo Magalhães | -11.9500000 | -46.0900000 |
| Brazil | *C. includens* | 2017 | Soybean | Bahia, BA | Correntina | -13.4802123 | -45.4089678 |
| Brazil | *C. includens* | 2017 | Soybean | Minas Gerais, MG | Uberlândia | -19.2164000 | -47.7494080 |
| Brazil | *C. includens* | 2017 | Soybean | Mato Grosso do Sul, MS | Campo Grande | -20.7010000 | -54.8300000 |
| Brazil | *C. includens* | 2017 | Soybean | Mato Grosso, MT | Sapezal | -13.6725000 | -58.8738889 |
| Brazil | *C. includens* | 2017 | Soybean | Mato Grosso, MT | Campo Verde | -15.3482768 | -54.9611493 |
| Brazil | *C. includens* | 2017 | Soybean | Paraná, PR | Campo Mourão | -24.1364880 | -52.4764840 |
| Brazil | *C. includens* | 2017 | Soybean | Paraná, PR | Rolândia | -23.3500000 | -51.5190000 |
| Brazil | *C. includens* | 2017 | Soybean | São Paulo, SP | Casa Branca | -21.8005523 | -47.0881334 |
| Brazil | *C. includens* | 2018 | Soybean | Bahia, BA | Luís Eduardo Magalhães | -12.1169444 | -45.9519444 |
| Brazil | *C. includens* | 2018 | Soybean | Goiás, GO | Rio Verde | -17.7393970 | -51.0385293 |
| Brazil | *C. includens* | 2018 | Soybean | Minas Gerais, MG | Uberlândia | -19.1164000 | -47.9494080 |
| Brazil | *C. includens* | 2018 | Soybean | Mato Grosso do Sul, MS | Campo Grande | -20.4680000 | -54.4160000 |
| Brazil | *C. includens* | 2018 | Soybean | Mato Grosso do Sul, MS | Campo Verde | -15.4910800 | -55.3478700 |
| Brazil | *C. includens* | 2018 | Soybean | Paraná, PR | Londrina | -23.1510000 | -51.1740000 |
| Brazil | *C. includens* | 2018 | Soybean | São Paulo, SP | Casa Branca | -21.5805200 | -47.0890300 |
| Brazil | *C. includens* | 2018 | Soybean | Rio Grande do Sul, RS | Não-Me-Toque | -28.3538100 | -52.7170900 |
| Brazil | *C. includens* | 2018 | Soybean | Mato Grosso, MT | Sapezal | -13.1543688 | -58.6523480 |
| Brazil | *C. includens* | 2018 | Soybean | Bahia, BA | Correntina | -13.4802123 | -45.4089678 |
| Brazil | *C. includens* | 2019 | Soybean | Bahia, BA | Roda Velha | -12.6750150 | -45.9665720 |
| Brazil | *C. includens* | 2019 | Soybean | Goiás, GO | Rio Verde | -17.7485116 | -50.5941897 |
| Brazil | *C. includens* | 2019 | Soybean | Mato Grosso, MT | Campo Verde | -15.5129475 | -55.2102624 |
| Brazil | *C. includens* | 2019 | Soybean | Paraná, PR | Campo Mourão | -24.0944325 | -52.3839866 |
| Brazil | *C. includens* | 2019 | Soybean | Paraná, PR | Londrina | -23.6031896 | -50.1427408 |
| Brazil | *C. includens* | 2020 | Soybean | Mato Grosso, MT | Campo Verde | -15.4965290 | -55.2841230 |
| Brazil | *C. includens* | 2020 | Soybean | Bahia, BA | Luís Eduardo Magalhães | -12.1498980 | -45.4588130 |
| Brazil | *C. includens* | 2020 | Soybean | Goiás, GO | Rio Verde | -17.8749110 | -50.0122370 |
| Brazil | *C. includens* | 2020 | Soybean | Mato Grosso, MT | Campo Grande | -20.6503580 | -54.5191060 |
| Brazil | *C. includens* | 2020 | Soybean | Paraná, PR | Londrina | -23.5062720 | -51.1307150 |
| Brazil | *C. includens* | 2021 | Soybean | Mato Grosso, MT | Campo Verde | -15.2828654 | -55.1481129 |
| Brazil | *C. includens* | 2021 | Soybean | Bahia, BA | Roda Velha | -13.1050350 | -46.1289782 |
| Brazil | *C. includens* | 2021 | Soybean | Paraná, PR | Campo Mourão | -23.8956040 | -52.3284600 |

Supporting Information (SI). Table 2. Populations of *C. includens* used in genotypic resistance monitoring (F_2_ screening).

| Country | Species | Year | Host | State | Location | Latitude | Longitude |
| --- | --- | --- | --- | --- | --- | --- | --- |
| Brazil | *C. includens* | 2017 | Soybean | Bahia, BA | Luís Eduardo Magalhães | -11.9500000 | -46.0900000 |
| Brazil | *C. includens* | 2017 | Soybean | Bahia, BA | Correntina | -13.4802123 | -45.4089678 |
| Brazil | *C. includens* | 2017 | Soybean | Minas Gerais, MG | Uberlândia | -19.2164000 | -47.7494080 |
| Brazil | *C. includens* | 2017 | Soybean | Mato Grosso do Sul, MS | Campo Grande | -20.7010000 | -54.8300000 |
| Brazil | *C. includens* | 2017 | Soybean | Mato Grosso, MT | Sapezal | -13.6725000 | -58.8738889 |
| Brazil | *C. includens* | 2017 | Soybean | Mato Grosso, MT | Campo Verde | -15.3482768 | -54.9611493 |
| Brazil | *C. includens* | 2017 | Soybean | Paraná, PR | Campo Mourão | -24.1364880 | -52.4764840 |
| Brazil | *C. includens* | 2017 | Soybean | Paraná, PR | Rolândia | -23.3500000 | -51.5190000 |
| Brazil | *C. includens* | 2017 | Soybean | Rio Grande do Sul, RS | Não-Me-Toque | -28.2500000 | -52.8500000 |
| Brazil | *C. includens* | 2017 | Soybean | São Paulo, SP | Casa Branca | -21.8005523 | -47.0881334 |
| Brazil | *C. includens* | 2018 | Soybean | Bahia, BA | Luís Eduardo Magalhães | -12.1169444 | -45.9519444 |
| Brazil | *C. includens* | 2018 | Soybean | Goiás, GO | Rio Verde | -17.7393970 | -51.0385293 |
| Brazil | *C. includens* | 2018 | Soybean | Minas Gerais, MG | Uberlândia | -19.1164000 | -47.9494080 |
| Brazil | *C. includens* | 2018 | Soybean | Mato Grosso do Sul, MS | Campo Grande | -20.4680000 | -54.4160000 |
| Brazil | *C. includens* | 2018 | Soybean | Goiás, GO | Cristalina | -16.9242300 | -47.6372500 |
| Brazil | *C. includens* | 2018 | Soybean | Mato Grosso do Sul, MS | Dourados | -22.7094400 | -55.0486100 |
| Brazil | *C. includens* | 2018 | Soybean | Mato Grosso, MT | Campo Verde | -15.4910800 | -55.3478700 |
| Brazil | *C. includens* | 2018 | Soybean | Paraná, PR | Campo Mourão | -24.1032368 | -52.3797550 |
| Brazil | *C. includens* | 2018 | Soybean | Paraná, PR | Ponta Grossa | -25.1412402 | -50.0816269 |
| Brazil | *C. includens* | 2018 | Soybean | Paraná, PR | Londrina | -23.1510000 | -51.1740000 |
| Brazil | *C. includens* | 2018 | Soybean | São Paulo, SP | Casa Branca | -21.5805200 | -47.0890300 |
| Brazil | *C. includens* | 2018 | Soybean | Rio Grande do Sul, RS | Não-Me-Toque | -28.3538100 | -52.7170900 |
| Brazil | *C. includens* | 2018 | Soybean | Rio Grande do Sul, RS | Bagé | -31.3280000 | -54.1070000 |
| Brazil | *C. includens* | 2019 | Soybean | Bahia, BA | Correntina | -13.7212760 | -46.1210570 |
| Brazil | *C. includens* | 2019 | Soybean | Bahia, BA | Luís Eduardo Magalhães | -11.7629420 | -45.7727840 |
| Brazil | *C. includens* | 2019 | Soybean | Bahia, BA | Roda Velha | -12.6750150 | -45.9665720 |
| Brazil | *C. includens* | 2019 | Soybean | Goiás, GO | Cristalina | -16.86651982 | -47.50627281 |
| Brazil | *C. includens* | 2019 | Soybean | Goiás, GO | Rio Verde | -17.7485116 | -50.5941897 |
| Brazil | *C. includens* | 2019 | Soybean | Maranhão, MA | Tasso Fragoso | -7.8260504 | -46.0062728 |
| Brazil | *C. includens* | 2019 | Soybean | Mato Grosso do Sul, MS | Campo Grande | -20.5806274 | -54.7336821 |
| Brazil | *C. includens* | 2019 | Soybean | Mato Grosso do Sul, MS | Maracaju | -20.7243184 | -54.5415967 |
| Brazil | *C. includens* | 2019 | Soybean | Mato Grosso, MT | Campo Verde | -15.5129475 | -55.2102624 |
| Brazil | *C. includens* | 2019 | Soybean | Paraná, PR | Campo Mourão | -24.0944325 | -52.3839866 |
| Brazil | *C. includens* | 2019 | Soybean | Paraná, PR | Londrina | -23.6031896 | -50.1427408 |
| Brazil | *C. includens* | 2019 | Soybean | Rio Grande do Sul, RS | Passo Fundo | -28.4948230 | -52.8051370 |
| Brazil | *C. includens* | 2019 | Soybean | São Paulo, SP | Casa Branca | -21.7545690 | -47.1245565 |
| Brazil | *C. includens* | 2019 | Cotton | Mato Grosso do Sul, MS | Chapadão do Sul | -18.7250700 | -52.5700990 |
| Brazil | *C. includens* | 2019 | Cotton | Mato Grosso, MT | Lucas do Rio Verde | -12.9763048 | -56.4589364 |
| Brazil | *C. includens* | 2019 | Cotton | Mato Grosso, MT | Rondonópolis | -16.8492717 | -54.0519069 |
| Brazil | *C. includens* | 2019 | Common bean | Bahia, BA | Luís Eduardo Magalhães | -12.1898544 | -45.4857939 |
| Brazil | *C. includens* | 2020 | Soybean | Mato Grosso, MT | Campo Verde | -15.4965290 | -55.2841230 |
| Brazil | *C. includens* | 2020 | Soybean | Bahia, BA | Luís Eduardo Magalhães | -12.1498980 | -45.4588130 |
| Brazil | *C. includens* | 2020 | Soybean | Goiás, GO | Rio Verde | -17.8749110 | -50.0122370 |
| Brazil | *C. includens* | 2020 | Soybean | Paraná, PR | Campo Mourão | -23.9618480 | -52.6301090 |
| Brazil | *C. includens* | 2020 | Soybean | Bahia, BA | Correntina | -14.0902040 | -45.9552980 |
| Brazil | *C. includens* | 2020 | Soybean | Mato Grosso do Sul, MS | Maracaju | -21.7142150 | -54.6296800 |
| Brazil | *C. includens* | 2020 | Soybean | Mato Grosso do Sul, MS | Campo Grande | -20.6503580 | -54.5191060 |
| Brazil | *C. includens* | 2020 | Soybean | Paraná, PR | Cascavel | -24.6582500 | -53.3568680 |
| Brazil | *C. includens* | 2020 | Soybean | Mato Grosso do Sul, MS | Chapadão do Sul | -18.7192870 | -52.5437350 |
| Brazil | *C. includens* | 2020 | Soybean | Goiás, GO | Cristalina | -16.8576200 | -47.6994550 |
| Brazil | *C. includens* | 2020 | Soybean | Paraná, PR | Londrina | -23.5062720 | -51.1307150 |
| Brazil | *C. includens* | 2020 | Soybean | Paraná, PR | Ponta Grossa | -24.9157420 | -50.2686850 |
| Brazil | *C. includens* | 2020 | Soybean | Maranhão, MA | Tasso Fragoso | -7.2323050 | -45.9542030 |
| Brazil | *C. includens* | 2020 | Soybean | Minas Gerais, MG | Uberlândia | -19.0833530 | -48.1684200 |
| Brazil | *C. includens* | 2020 | Soybean | Rio Grande do Sul, RS | Bagé | -31.1990560 | -54.0296290 |
| Brazil | *C. includens* | 2020 | Cotton | Bahia, BA | Correntina | -13.0217350 | -46.2134100 |
| Brazil | *C. includens* | 2020 | Cotton | Bahia, BA | Luís Eduardo Magalhães | -12.0222880 | -45.7589750 |
| Brazil | *C. includens* | 2020 | Cotton | Mato Grosso do Sul, MS | Chapadão do Sul | -18.6824560 | -52.9155419 |
| Brazil | *C. includens* | 2021 | Soybean | Mato Grosso, MT | Campo Verde | -15.2828654 | -55.1481129 |
| Brazil | *C. includens* | 2021 | Soybean | Goiás, GO | Cristalina | -17.0477980 | -47.5754300 |
| Brazil | *C. includens* | 2021 | Soybean | Mato Grosso, MT | Sapezal | -13.5792291 | -56.0666686 |
| Brazil | *C. includens* | 2021 | Soybean | Bahia, BA | Correntina | -13.9314721 | -46.1749146 |
| Brazil | *C. includens* | 2021 | Soybean | Paraná, PR | Cascavel | -24.9129670 | -53.5483930 |
| Brazil | *C. includens* | 2021 | Soybean | Paraná, PR | Londrina | -23.0921135 | -51.0906915 |
| Brazil | *C. includens* | 2021 | Soybean | Mato Grosso do Sul, MS | Maracaju | -21.6476179 | -55.0930083 |
| Brazil | *C. includens* | 2021 | Soybean | Minas Gerais, MG | Uberlândia | -18.58878077 | -48.67458462 |
| Brazil | *C. includens* | 2021 | Soybean | Goiás, GO | Rio Verde | -17.94815700 | -50.42512700 |
| Brazil | *C. includens* | 2021 | Soybean | Paraná, PR | Campo Mourão | -23.89560400 | -52.32846000 |
| Brazil | *C. includens* | 2021 | Soybean | Rio Grande do Sul, RS | Passo Fundo | -28.33136587 | -52.46384514 |
| Brazil | *C. includens* | 2021 | Soybean | Mato Grosso do Sul, MS | Campo Grande | -20.46455500 | -54.84998700 |
| Brazil | *C. includens* | 2021 | Soybean | São Paulo, SP | Conchal | -22.3737352 | -47.1769073 |
| Brazil | *C. includens* | 2021 | Soybean | Mato Grosso, MT | Lucas do Rio Verde | -13.06724078 | -55.86105386 |
| Brazil | *C. includens* | 2021 | Soybean | Bahia, BA | Roda Velha | -13.10503499 | -46.12897823 |
| Brazil | *C. includens* | 2021 | Soybean | Maranhão, MA | Tasso Fragoso | -7.212587950 | -45.88129908 |

Supporting Information (SI). Table 3. Populations of *C. includens*, *R. nu* and *C. aporema* used in leaf-disc bioassays or complementation test.

| Country | Species | Year | Host | State | Location | Latitude | Longitude |
| --- | --- | --- | --- | --- | --- | --- | --- |
| Argentina | *C. includens* | 2019 | Soybean | Buenos Aires | Balcarce | -37.8977889 | -58.3042194 |
| Argentina | *C. includens* | 2019 | Soybean | Santiago del Estero | Bandera | -28.8238271 | -62.2524799 |
| Argentina | *C. includens* | 2019 | Soybean | Santiago del Estero | Bobadal | -26.6807880 | -64.0972260 |
| Argentina | *C. includens* | 2019 | Soybean | Entre Ríos | Concordia | -31.2031724 | -58.3364676 |
| Argentina | *C. includens* | 2019 | Soybean | Santa Fe | Margarita | -29.7078848 | -60.1443154 |
| Argentina | *C. includens* | 2019 | Soybean | Santa Fe | Reconquista | -29.1049807 | -59.8052415 |
| Argentina | *C. includens* | 2019 | Soybean | Tucumán | San Agustín | -26.8742461 | -64.8306549 |
| Argentina | *C. includens* | 2019 | Soybean | - | Lab Susceptible | - | - |
| Argentina | *C. includens* | 2020 | Soybean | Santiago del Estero | Bandera | -28.7680280 | -62.1825400 |
| Argentina | *C. includens* | 2020 | Soybean | - | Lab Susceptible | - | - |
| Argentina | *C. includens* | 2020 | Soybean | Salta | Las Lajitas | -24.8122340 | -64.1144420 |
| Argentina | *C. includens* | 2020 | Soybean | Catamarca | Los Altos | -27.9499320 | -65.4570470 |
| Argentina | *C. includens* | 2020 | Soybean | Córdoba | Marcos Juárez | -32.7717180 | -62.3377820 |
| Argentina | *C. includens* | 2020 | Soybean | Santa Fe | Margarita | -29.7255690 | -60.0449670 |
| Argentina | *C. includens* | 2020 | Soybean | Santiago del Estero | Nueva Esperanza | -26.1659290 | -63.9879280 |
| Argentina | *C. includens* | 2020 | Soybean | Chaco | San Bernardo | -27.3112500 | -60.7015280 |
| Argentina | *C. includens* | 2020 | Soybean | Tucumán | Tala Pozo | -26.7248600 | -64.7496790 |
| Brazil | *C. includens* | 2019 | Soybean | Bahia | Correntina | -13.0217350 | -46.2134100 |
| Brazil | *C. includens* | 2019 | Common bean | São Paulo | Guaíra | -20.3271000 | -48.3359000 |
| Brazil | *C. includens* | 2019 | Soybean | Bahia | Luís Eduardo Magalhães | -12.0222880 | -45.7589750 |
| Brazil | *C. includens* | 2019 | Soybean | - | Lab Susceptible | - | - |
| Brazil | *C. includens* | 2020 | Soybean | Bahia | Correntina | -13.9314271 | -46.1749146 |
| Argentina | *R. nu* | 2019 | Soybean | Entre Ríos | Concordia | -31.2031724 | -58.3364676 |
| Argentina | *R. nu* | 2019 | Soybean | Buenos Aires | Acevedo | -33.7846939 | -60.4721805 |
| Argentina | *R. nu* | 2019 | Soybean | Tucumán | Burruyacú | -26.4150370 | -64.6997860 |
| Argentina | *R. nu* | 2019 | Soybean | Córdoba | Marcos Juárez | -32.7158508 | -62.1335987 |
| Argentina | *R. nu* | 2019 | Soybean | Santa Fe | Margarita | -29.7078848 | -60.1443154 |
| Argentina | *R. nu* | 2019 | Soybean | Santiago del Estero | Pozo Hondo | -27.1785275 | -64.4189680 |
| Argentina | *R. nu* | 2019 | Soybean | San Luis | Quines | -32.0667014 | -65.8395463 |
| Argentina | *R. nu* | 2019 | Soybean | - | Lab Susceptible | - | - |
| Argentina | *R. nu* | 2020 | Soybean | Buenos Aires | Acevedo | -33.7846940 | -60.4721810 |
| Argentina | *R. nu* | 2020 | Soybean | - | Lab Susceptible | - | - |
| Argentina | *R. nu* | 2020 | Soybean | Santa Fe | Margarita | -29.7255690 | -60.0449670 |
| Argentina | *R. nu* | 2020 | Soybean | Chaco | Villa Angela | -27.5413390 | -60.7586360 |
| Argentina | *R. nu* | 2020 | Soybean | Buenos Aires | Balcarce | -37.8977890 | -58.3042190 |
| Argentina | *R. nu* | 2020 | Soybean | Salta | Las Lajitas | -25.0650400 | -64.1839940 |
| Argentina | *R. nu* | 2020 | Soybean | Córdoba | Marcos Juárez | -32.7717190 | -62.3377830 |
| Argentina | *R. nu* | 2020 | Soybean | Santiago del Estero | Nueva Esperanza | -26.1659290 | -63.9879280 |
| Argentina | *R. nu* | 2020 | Soybean | Chaco | Roque Sáenz Peña | -26.7009100 | -60.3693800 |
| Argentina | *R. nu* | 2020 | Soybean | Tucumán | San Agustín | -26.8742460 | -64.8306550 |
| Argentina | *R. nu* | 2020 | Soybean | Santa Fe | Venado Tuerto | -33.6188230 | -61.8920540 |
| Brazil | *R. nu* | 2020 | Soybean | São Paulo | Paranapanema | -23.4016540 | -48.7295100 |
| Brazil | *R. nu* | 2020 | Soybean | São Paulo | Taquarituba | -23.5312810 | -49.2585510 |
| Brazil | *R. nu* | 2020 | Soybean | Minas Gerais | Uberaba | -19.2397140 | -47.7389110 |
| Brazil | *R. nu* | 2021 | Soybean | Minas Gerais | Perdizes | -19.3732350 | -47.3423440 |
| Brazil | *R. nu* | 2021 | Soybean | São Paulo | Taquarituba | -23.6248520 | -49.1899590 |
| Brazil | *C. aporema* | 2021 | Soybean | São Paulo, SP | Itararé | -24.0280520 | -49.3590880 |
| Brazil | *C. aporema* | 2021 | Soybean | Minas Gerais, MG | Perdizes | -19.3732350 | -47.3423440 |
| Brazil | *C. aporema* | 2021 | Soybean | Goiás, GO | Cristalina | -16.1780530 | -47.4572470 |
| Brazil | *C. aporema* | 2021 | Soybean | Paraná, PR | Tibagi | -24.4399160 | -50.2693740 |


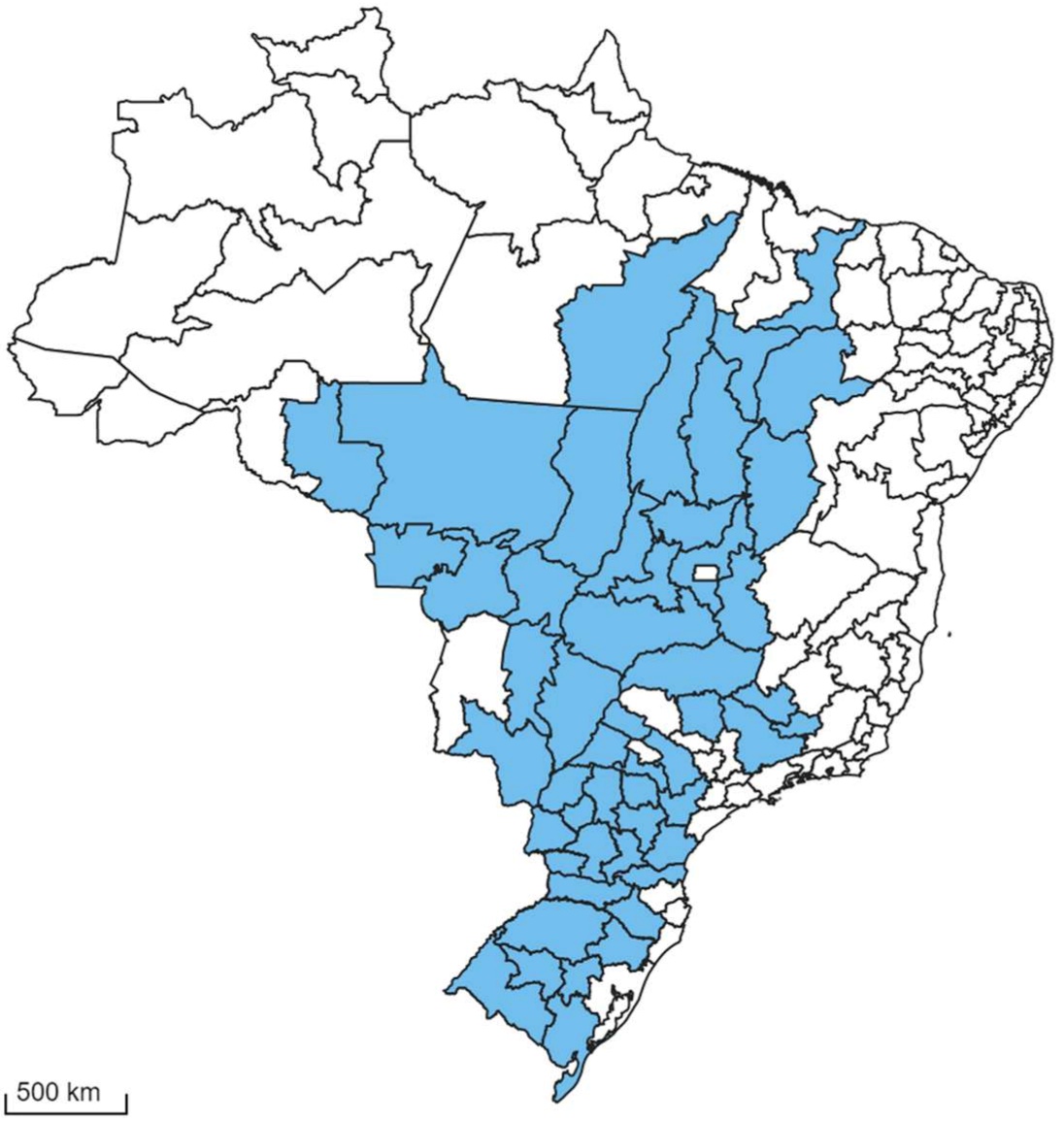


Supporting Information (SI). Figure 1. Brazilian mesoregions used for refuge compliance analysis. Map was generated using TIBCO Spotfire Analyst 10.10.2 LTS <https://www.tibco.com/products/tibco-spotfire>.
